# Neuroinflammation after intracerebral hemorrhage

**DOI:** 10.3389/fncel.2014.00388

**Published:** 2014-11-20

**Authors:** Eva Mracsko, Roland Veltkamp

**Affiliations:** ^1^Department of Neurology, University HeidelbergHeidelberg, Germany; ^2^Division of Brain Sciences, Imperial CollegeLondon, UK

**Keywords:** stroke, intracerebral hemorrhage, neuroinflammation, innate immunity, adaptive immunity

## Abstract

Spontaneous intracerebral hemorrhage (ICH) is a particularly severe type of stroke for which no specific treatment has been established yet. Although preclinical models of ICH have substantial methodological limitations, important insight into the pathophysiology has been gained. Mounting evidence suggests an important contribution of inflammatory mechanisms to brain damage and potential repair. Neuroinflammation evoked by intracerebral blood involves the activation of resident microglia, the infiltration of systemic immune cells and the production of cytokines, chemokines, extracellular proteases and reactive oxygen species (ROS). Previous studies focused on innate immunity including microglia, monocytes and granulocytes. More recently, the role of adaptive immune cells has received increasing attention. Little is currently known about the interactions among different immune cell populations in the setting of ICH. Nevertheless, immunomodulatory strategies are already being explored in ICH. To improve the chances of translation from preclinical models to patients, a better characterization of the neuroinflammation in patients is desirable.

## Introduction

Intracerebral hemorrhages (ICH) account for 10–15% of all strokes (Qureshi et al., 2009). It is a particularly severe stroke subtype that is associated with a mortality rate of 30–50%. Moreover, 74% of the survivors remain functionally dependent 12 months after the ictus (van Asch et al., 2010). Currently, the overall incidence of ICH is 24.6 per 100,000 person per year (van Asch et al., 2010) but incidence is expected to have doubled by 2050 (Qureshi et al., 2001) due to aging and the spreading use of anticoagulants (Wang, 2010). Intracerebral hemorrhages is strongly associated with cerebral microvascular diseases (Xi et al., 2006). The most frequent underlying disorder is hypertensive microangiopathy which predominantly manifests in deep cerebral structures (basal ganglia, brain stem and cerebellum) (Fisher, 1971, 2003). In the elderly, cerebral amyloid angiopathy develops in cortical arteriolar and venular microvessels (Thanvi and Robinson, 2006). Intracerebral hemorrhages in association with the use of oral anticoagulants is increasingly frequently encountered. Hemorrhage originating from aneurysms or vascular malformations is less frequent (Qureshi et al., 2001). Risk factors for ICH include genetic variants of apolipoprotein E, ethnic differences and life style factors such as smoking and alcohol intake (O’Donnell et al., 2010). Several determinants of outcome have been clearly identified. Predictors of poor clinical outcome are the initial hematoma volume, hematoma expansion during the first day, location of the hematoma, extent of brain edema, age and neurological status on admission (Hanley, 2009; Mendelow et al., 2011; Kuramatsu et al., 2013). Although several of these factors are potentially modifiable, no effective medical or surgical therapy has been firmly established for acute ICH beyond treatment in dedicated stroke and critical care units (Steiner et al., 2006, 2014; Xi et al., 2006; Morgenstern et al., 2010; Keep et al., 2012). Current efforts in clinical trials focus on blood pressure control (Anderson et al., 2013), modified surgical approaches or hemostasis in selected patients (Mayer et al., 2008; Morgan et al., 2008; Newell et al., 2011; Ziai et al., 2014). Despite the lack of evidence from randomized clinical trials, specialized neurovascular centers offer medical and surgical therapies for selected patients but otherwise ICH therapy remains supportive within a framework of general critical care management (Kuramatsu et al., 2013).

The need for new therapeutic approaches for ICH has prompted a search for the molecular and cellular mechanisms that underlie early and delayed brain damage after ICH. Clearly, several research themes are shared with other acute and chronic degenerative brain disorders. However, the appearance of extracellular blood in the brain, that results in the release of the hemoglobin constituents heme and iron, triggers specific pathophysiological cascades or modifies the timing of other processes.

In particular, there is increasing evidence that inflammatory mechanisms participate in early and delayed phases after ICH. After reviewing some limitations of preclinical modeling of ICH, the present review will summarize the evidence supporting an essential role of inflammation to brain damage and potential repair after ICH.

## Preclinical models of ICH

Animal models of ICH have been established in many different species (for review see James et al., 2008). A major limitation of most models is that an invasive procedure is required to induce the hemorrhage that inadvertently implies a limited brain trauma. The most frequently used methods and species, respectively, are the intracerebral injection of autologous blood or bacterial collagenase in rodents (MacLellan et al., 2008, 2010). Although both models are suitable to induce hematomas of various sizes and location, the differences between these models may influence the pathomechanisms of ICH and the neurological outcome.

Injection of autologous blood (Bullock et al., 1984) creates a single large bleeding and allows studying the mechanisms of hemorrhage-induced neuronal damage. However, it fails to reproduce the aspect of continuing bleeding and hematoma expansion. Secondary hematoma enlargement occurs in about 1/3 of patients during the first day after ICH and is an important predictor of poor neurological outcome (Brott et al., 1997; Davis et al., 2006). In contrast, injection of bacterial collagenase (Rosenberg et al., 1990) dissolves the basal lamina of small cerebral blood vessels and results in continuous parenchymal bleeding for several hours (MacLellan et al., 2008). However, the vascular source of bleeding in the collagenase model differs from most human ICH in which bleeding is of penetrating arteriolar origin (Clark et al., 1998; Wang et al., 2003; Tang et al., 2004). Another disadvantage of this model is that higher doses of collagenase can induce direct neurotoxicity (Matsushita et al., 2000; Chu et al., 2004) which may complicate the interpretation of results with neuroprotective strategies.

The size of the hematoma, which determines outcome both in man (Broderick et al., 1993) and in rodents (MacLellan et al., 2006), can be varied in both ICH-models by changing the injected blood volume or collagenase dose. However, injection of a higher blood volume may produce difficulties by the injected blood spreading along the corpus callosum or flowing back through the needle insertion canal. These problems can be reduced by using the double injection method (Belayev et al., 2003), where a small amount of blood is allowed to clot and followed by the injection of the remaining blood volume. In comparison with the blood injection model where the tissue is split apart by the hematoma, collagenase induces a less dense hemorrhage which infiltrates the parenchyma (MacLellan et al., 2008) resulting in bigger hematoma volume in case of matched blood content between the two models (Mracsko et al., 2014).

In both models, macroscopic hematoma size decreases already during the first days after surgery (Mracsko et al., 2014), and the hematoma resolves completely in about 21 days (Zhao et al., 2007). In contrast, the resolution of the hematoma takes several weeks in patients and usually leaves a cavity in the brain with focal atrophy and ventricular enlargement (Dolinskas et al., 1977).

In conclusion, both ICH methods have their advantages and limitations. These differences should be carefully considered when choosing a model to address the outcome parameters of interest and when interpreting the findings.

## Deleterious mechanical effects of the hematoma

**Figure 1:**
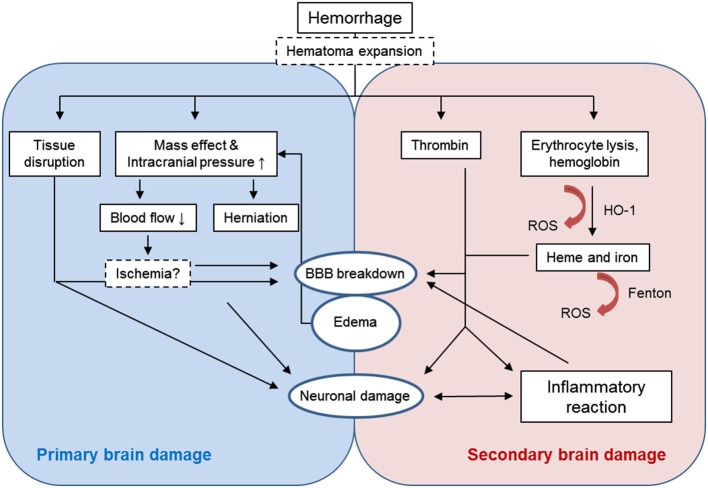
**Primary and secondary brain damage after intracerebral hemorrhage**.

Primary brain injury after ICH is caused by the tissue disruption due to parenchymal blood accumulation and the mechanical damage associated with the mass effect (Figure 1). Besides treating increased intracranial pressure (Helbok et al., 2011), surgical interventions to remove the blood clot and release the pressure would appear a plausible approach in this phase (Gautschi and Schaller, 2013). In about one third of patients (Kazui et al., 1996; Brott et al., 1997), re-bleeding and the expansion of the hemorrhage within the first day after the ictus further exacerbates the mass effect and thus neurological damage. Preventing this complication by aggressive antihypertensive therapy or by administration of hemostatic factors may prevent secondary hematoma growth. (Sakamoto et al., 2013). However, evidence for clinical efficacy is limited. The concept of brain damage resulting from peri-hematomal ischemia induced by the increased intracranial pressure has not been confirmed in studies using positron emission tomography in patients (Zazulia et al., 2001). However, a recent magnetic resonance imaging (MRI) study found ischemic events in one third of ICH patients within 1 month after the ictus (Menon et al., 2012).

Immediately after ICH, peri-hematomal edema develops which increases intracranial pressure and contributes to the mass effect (Xi et al., 2006). Edema in ICH is associated with higher in-hospital mortality (Staykov et al., 2011). In animal models, edema peaks already 3–4 days after ICH-induction (Xi et al., 1998). In contrast, the edema expands in ICH patients until at least 10 days after the ictus (Staykov et al., 2011). In the first hours after ICH, edema is mainly formed by plasma egress due to the increased hydrostatic pressure and the damaged blood-brain barrier (BBB); edema also results from extruded serum during clot retraction (Wagner et al., 1996). Later on, thrombin production, erythrocyte lysis and the triggered inflammatory processes are responsible for edema formation (Xi et al., 2001a).

## Mechanisms of secondary brain damage

Besides the mechanical tissue damage caused by the initial hematoma, injured brain cells and the extravasated components of the blood clot trigger a complex sequence of parallel and sequential deleterious mechanisms including inflammatory and oxidative stress pathways (Aronowski and Zhao, 2011; Figure 1).

Activation of hemostatic mechanisms is a physiological tissue response to hemorrhage to stop the bleeding. Thrombin is essential for the blood coagulation processes and gets activated within the first hour after ICH (Gong et al., 2008). Intracerebral injection of thrombin leads to early brain edema formation by direct opening of the BBB (Lee et al., 1997) and to neuronal damage at days 1 and 3 after ICH (Gong et al., 2008). High concentrations of thrombin induce neuronal damage *in vitro*, however, low concentrations are neuroprotective against various insults including ischemia or oxidative stress (Vaughan et al., 1995; Donovan et al., 1997; Striggow et al., 2000). Moreover, thrombin has an important role in brain recovery after intracerebral hemorrhage (Hua et al., 2009) possibly via the initiation of neurogenesis (Yang et al., 2008) and angiogenesis (Tarzami et al., 2006; Tsopanoglou and Maragoudakis, 2007). Therefore, the role of thrombin after ICH remains controversial (Xi et al., 2003, 2006; Keep et al., 2012).

The lysis of erythrocytes within the first days after ICH leads to the release of hemoglobin which is then converted by the heme oxygenase-1 enzyme (HO-1) into neurotoxic components such as heme and iron which are major contributors to secondary brain injury (Wagner et al., 2003; Wu et al., 2003; Keep et al., 2012). Intracerebral injection of lyzed erythrocytes or hemoglobin and iron result in brain edema formation and neuronal damage (Xi et al., 1998; Huang et al., 2002). The proposed mechanism of heme- and iron-induced neurotoxicity is the induction of oxidative stress due to the activity of HO-1 (Koeppen et al., 2004; Wang and Doré, 2007a) and the iron-mediated free radical production via the Fenton-reaction (Wu et al., 2003, 2011; Clark et al., 2008).

The inflammatory reaction comprising both cellular and molecular components is a common response of the central nervous system (CNS) to various stimuli. Neuroinflammation after ICH involves the early activation of resident microglia, release of proinflammatory mediators and the influx of peripheral leukocytes and has major role in the pathophysiology of secondary brain damage (Wang and Doré, 2007b; Wang, 2010). Components of both innate and adaptive immune system take part of ICH-induced neuroinflammation. At present, the involvement of antigen specific immune processes remains unclear in both ischemic and hemorrhagic stroke (Iadecola and Anrather, 2011).

## Microglia/macrophages

The first activated innate immune cells are microglia which reside in the CNS. They continuously scan the extracellular brain environment and can be activated within minutes after tissue damage. Danger-associated molecular patterns including ATP, neurotransmitters, nucleic acids, heat shock proteins and high mobility group box 1 are released to the extracellular space from necrotic neurons after ICH (Ohnishi et al., 2011). These stimuli act on distinct microglia receptors including Toll-like receptors (TLRs) and the receptor of advanced glycosylation end-products (Taylor and Sansing, 2013). Several TLRs including TLR4 are involved in the neuroinflammatory processes after ICH (Fang et al., 2013). TLR4 is predominantly expressed in CD11b+ microglial cells and is upregulated early after ICH subsequently leading to the upregulation of proinflammatory genes via nuclear factor-κB (NF-κB) signaling (Teng et al., 2009; Lin et al., 2012). Besides danger signals originating from damaged neuronal cells, blood components such as thrombin, fibrin and heme can also trigger inflammatory processes through the TLR/NF-κB pathway (Loftspring et al., 2010; Lin et al., 2012; Wang et al., 2014). Hemoglobin triggers an inflammatory response via assembly of TLR2/TLR4 heterodimers (Wang et al., 2014). Experimental and clinical data suggest that TLR4 contributes to neuronal damage in ICH. TLR4 deficiency (Teng et al., 2009; Sansing et al., 2011b; Lin et al., 2012) or blockade (Wang et al., 2013) lowers brain water content (i.e., edema) and reduces neurological deficit. In patients, higher expression of TLR2 and TLR4 in monocytes on admission was independently associated with poor outcome (Rodríguez-Yáñez et al., 2012). Therefore, antagonization of the TLR4 signaling may represent a therapeutic target after ICH.

Besides TLRs, microglia can be activated by thrombin via the proteinase activated receptor-1 (PAR-1) and mitogen-activated protein kinase signaling pathways (Fujimoto et al., 2007; Ohnishi et al., 2007). This leads to increased production of tumor necrosis factor-α (TNF-α) and neuronal death (Ohnishi et al., 2010). Microglia endocytose erythrocyte remnants via scavenger receptors such as CD36, which initiates microglial activation (Aronowski and Zhao, 2011; Fang et al., 2014).

Upon stimulation, microglia cells will be rounded gaining an ameboid appearance and high phagocytic activity (Kreutzberg, 1996). They are difficult to distinguish from activated macrophages which express the same cellular surface markers including CD11b, Iba-1, isolectin B4 (Ginhoux et al., 2010). However, multi-parameter characterization by flow cytometry allows the definition of microglia population as CD45^low^/CD11b+ cells (Campanella et al., 2002; D’Mello et al., 2009; Parney et al., 2009; Gabrusiewicz et al., 2011; Patel et al., 2013; Mracsko et al., 2014; Tang et al., 2014). Both macrophages and microglia can have either the classically activated M1 or the alternatively activated M2 phenotype (Kigerl et al., 2009; David and Kroner, 2011). M1 polarized microglia produce proinflammatory, largely deleterious cytokines such as TNF-α, interleukin-1β (IL-1β) or IL-6 and pro-oxidant enzymes such as inducible nitric oxide synthase (Liao et al., 2012; Kobayashi et al., 2013). In contrast, M2 polarized microglia have arginase activity, produce neurotrophic factors and IL-10. The M2 microglia phenotype has been associated with neuroprotective and regenerative effects after brain injury (Ponomarev et al., 2007). Due to this polarity, microglia/macrophage can exert controversial effects in brain diseases and injuries (Taylor and Sansing, 2013).

Microglial activation takes place in various neurological disorders including CNS and peripheral infections, neurodegenerative diseases, traumatic brain injury, ischemic and hemorrhagic stroke (Suzuki et al., 2011; Püntener et al., 2012; Hernandez-Ontiveros et al., 2013; Patel et al., 2013; Taylor and Sansing, 2013; Doens and Fernández, 2014). Besides the clearance of cell debris, microglia play also an important role in the phagocytosis of blood components released into the brain parenchyma (Aronowski and Zhao, 2011). In experimental ICH, microglial activation occurs as early as 1 h following collagenase injection (Wang and Doré, 2007a) and 4 h after autologous blood injection (Xue and Del Bigio, 2000). The number of activated microglia/macrophages peaks at 72 h and returns to normal level 3–4 weeks after ICH (Wang, 2010; Yabluchanskiy et al., 2010; Sansing et al., 2011b).

Upon various stimuli, microglia and brain macrophages produce proinflammatory cytokines including TNF-α and IL-1β (Wang and Doré, 2007b), chemokines (Matsushita et al., 2014) and reactive oxygen species (ROS; Yang et al., 2014a). Beyond the neurotoxic cytokines, chemokines such as CXCL2 produced by microglia (Shiratori et al., 2010) have chemotactic activity on neutrophils and thus exacerbate the inflammatory reaction (Tessier et al., 1997). Moreover, M1 polarized microglia create microglia-T cell crosstalk due to antigen presentation via MHCII expression (Starossom et al., 2012). Thus, microglia also enforce early neuroinflammation by recruiting and activating blood-derived leukocytes which may worsen ICH-induced neuronal damage.

On the other hand, microglia play a key role in hematoma resolution and therefore in the recovery phase after ICH. A more effective and faster clearance of intracerebral blood could limit the inflammatory processes that are triggered by blood constituents in the brain parenchyma (Zhao et al., 2007). Moreover, the chemokine receptor CX3CR1 is required for M2 polarization of microglia facilitating recovery after ICH (Taylor and Sansing, 2013).

The essential pathophysiological role of microglia/macro­phages after ICH suggests a therapeutic potential. On the other hand, microglial functions are diverse and cannot be classified as either good or bad. Moreover, different microglial subsets may send opposing signals, and predominant functional effects may differ depending on timing after the event (cp ischemia; Lalancette-Hébert et al., 2007).

In experimental ICH, blockade of TLR4 reduced neuronal loss and edema formation and improved neurological function. The effects resulted from inhibition of downstream signaling mechanisms and the lower expression of proinflammatory cytokines (Wang et al., 2013). In another study, the TLR4 inhibitor TAK-242 upregulated CD36 scavenger receptor expression thereby promoting faster hematoma resolution and attenuating neurological deficit (Fang et al., 2014). Minocycline is a tetracycline-based molecule which can inhibit microglia activation (Tikka and Koistinaho, 2001). Minocyclin has been tested in numerous studies to moderate neuronal damage after ICH. Minocycline reduces thrombin-induced microglial TNF-α and IL-1β expression *in vitro* (Wu et al., 2009). In the same study, minocycline reduced brain edema 3 days after intracerebral blood injection, diminished neurological deficit and decreased brain atrophy 28 days following ICH (Wu et al., 2010). These effects were accompanied by reduced numbers of microglia/macrophages around the hematoma 5 days following ICH (Szymanska et al., 2006). Others found preserved microvessels along with reduced brain water content, and lower levels of TNF-α and matrix metalloproteinase-12 (MMP-12) in minocycline-treated rats (Wasserman and Schlichter, 2007). In these studies, the treatment was applied from up to 6 h after induction of ICH suggesting clinical relevance. As a consequence, a randomized,-single-blinded clinical trial of minocycline in ICH has been initiated (A Pilot Study of Minocycline in Intracerebral Hemorrhage Patients (MACH); NCT01805895). Further molecules targeting microglia activation and function have been recently tested after ICH. The mitogen-activated protein kinase inhibitor sesamin (Ohnishi et al., 2013), as well as sinomenine (Yang et al., 2014a) and curcumin (Yang et al., 2014c) with anti-inflammatory and anti-oxidant properties were neuroprotective in ICH. However, their distinct mechanisms of action require further investigation.

Hematoma resolution by microglia/macrophages has also been recognized as a therapeutic target after ICH. Peroxisome proliferator-activated receptor-γ agonist induced CD36-mediated clearance of erythrocytes by microglia *in vitro*. It promoted hematoma resolution, reduced neuronal loss and neurological deficit *in vivo* (Zhao et al., 2007, 2009). Therefore, besides blocking the acute detrimental effects of microglia activation, stimulating microglial phagocytosis and thus enhancing recovery may also have therapeutic potential.

## BBB breakdown and invasion of systemic immune cells

The physical BBB is formed by capillary endothelial cells, which are connected via tight junctions resulting in very low permeability. Besides endothelial cells, perivascular cells such as pericytes and astrocytes and the extracellular matrix have an important regulatory role on BBB function. Increased permeability of the BBB can be caused by changes in the para- and transcellular routes or by disruption of the extracellular matrix (Keep et al., 2014; Knowland et al., 2014). In ischemic stroke, BBB dysfunction results from insufficient oxygen and glucose supply (Ronaldson and Davis, 2012). In contrast, the absence of ICH-induced ischemic damage (Zazulia et al., 2001) suggests that other mechanisms induce BBB hyperpermeability in ICH. Thrombin has been shown to induce BBB disruption via proteinase activated receptor-1 mediated mechanisms (Liu et al., 2010). Hemoglobin itself and its degradation products heme and iron also increase permeability of the BBB (Yang et al., 2013). Accordingly, the iron chelator deferoxamine (Nakamura et al., 2004; Okauchi et al., 2010) and HO inhibitors (Gong et al., 2006) reduce ICH-induced brain edema.

Matrix metalloproteinases belong to the group of endopeptidases just as other proteases like serine or cysteine proteases. They have important role in the remodeling of extracellular matrix but under inflammatory conditions activation of MMPs results in BBB dysfunction, increased capillary permeability and brain edema formation after ICH (Rosenberg and Navratil, 1997). Matrix metalloproteinases have been intensively studied in ICH in the last two decades and the available information on their role in ICH has been reviewed in detail (Wang and Doré, 2007b; Florczak-Rzepka et al., 2012). Although inhibition of MMPs may decrease ICH-induced brain injury, MMPs also have an important role in the regulation of neurogenesis, myelin function and axonal growth (Pepper, 2001; Kaczmarek et al., 2002; Cunningham et al., 2005). Therefore rather the modulation than long-term inhibition of MMPs may be considered for ICH treatment.

The strict regulation of the immune cell infiltration into the brain parenchyma through the immunological BBB plays an important role in the immune privilege of the CNS (Pachter et al., 2003). During neuroinflammatory processes, the expression of adhesion molecules on leukocytes and of their ligands on endothelial cells in postcapillary venules increases. As a consequence, leukocytes adhere to the wall of these venules. Infiltration through the BBB involves rolling, adhesion and transendothelial migration of leukocytes. Adhesion molecules that participate in this process are classified into three types: selectins, the superfamily of immunoglobulins and the integrins (Brea et al., 2009; Iadecola and Anrather, 2011). The expression of intracellular adhesion molecule-1 is upregulated already hours after ICH (Gong et al., 2000; Yang et al., 2011). The vascular adhesion protein-1 has been also shown to be upregulated after ICH, and its inhibitors reduced neutrophil invasion and brain damage (Ma et al., 2011).

The brain infiltrating leukocytes produce proinflammatory cytokines and MMPs leading to further disruption of the BBB (Xi et al., 2006; Wang and Doré, 2007b; Aronowski and Zhao, 2011). Therefore, peripheral leukocytes and the BBB are in tight reciprocal connection that makes it difficult to evaluate the effect of distinct compounds on BBB integrity. Essentially, any compound that influences the ICH-induced inflammatory reaction also affects BBB integrity and vica versa.

In experimental and clinical ICH blood-borne leukocytes invade the hemorrhagic brain (Lee et al., 1975; Del Bigio et al., 1996; Gong et al., 2000; Xue and Del Bigio, 2000; Mayne et al., 2001a; Peeling et al., 2001; Wang and Tsirka, 2005). In principle, leukocytes found in the brain after ICH could originate from the inflowing blood in the hematoma. Alternatively, systemic immune cells may actively migrate across the BBB to enter the brain (Xi et al., 2006). The origin of leukocytes located in the brain after ICH can be determined by using the leukocyte marker CD45.1 transgenic mice in blood injection models (Sansing et al., 2011b; Hammond et al., 2012; Mracsko et al., 2014). In this approach, blood from CD45.2 expressing wild type mice is injected into the brain of CD45.1 expressing mice or vice versa and brain located leukocytes are analyzed by flow cytometry for CD45.2 and CD45.1 expression. These studies agree that already 1 day after blood injection the majority of leukocytes isolated from the brain originates from the blood circulation rather than from the injected blood. A methodological limitation of this approach is that the traumatic injury caused by the insertion of the injection needle alone results in a relatively high number of infiltrating leukocyte in sham operated animals (Loftspring et al., 2009; Mracsko et al., 2014). Therefore, differences of injection techniques and even needles can result in discrepancies between different workgroups regarding infiltrating cell numbers.

## Monocytes

Monocytes are produced by bone marrow from monoblasts and mature into different types of macrophages. In the CNS the renewal of the microglia cell population takes place by local expansion and, at lower rate, by replenishment by circulating monocytes (Ajami et al., 2007). As mentioned above, the distinction of infiltrating monocyte/macrophages from microglia is difficult due to the identical surface activation markers they express. To distinguish the roles and distributions of microglia and peripheral monocytes, several studies on cerebral ischemia used bone marrow chimeric mice generated by transplanting green fluorescent protein transgenic bone marrow into irradiated wild-type recipients (Schilling et al., 2003, 2005; Tanaka et al., 2003). So far the application of this approach in ICH is limited to one study (Hammond et al., 2014). Taking advantage of differential CD45 expression between microglia and monocyte/macrophages, flow cytometry studies have also differentiated infiltrating monocytes (Sansing et al., 2011a,b; Hammond et al., 2012; Mracsko et al., 2014). Monocytes invade already within 12 h after ICH outnumbering the neutrophil population (Hammond et al., 2012), and their number peaks by day 5 (Mracsko et al., 2014). Monocyte infiltration is reduced after neutrophil depletion (Sansing et al., 2011a) or in TLR4 deficiency (Sansing et al., 2011b). The monocyte chemoattractant protein-1 and its receptor CC chemokine receptor 2 (CCR2) are involved in the migration of monocytes into the hemorrhagic brain (Yao and Tsirka, 2012b). Monocyte chemoattractant protein-1 is elevated in the brain 24 h after experimental ICH (Chang et al., 2011; Ma et al., 2011) as well as in the serum of patients associated with poor functional outcome 7 days following ICH (Hammond et al., 2014). Accordingly, chimeric mice with wild type CNS and CCR2 deficiency exhibit attenuated motor dysfunction after ICH (Hammond et al., 2014). At the same time, CCR2+ inflammatory monocytes seem to be important regulators of hematoma clearance and functional recovery after ICH (Yao and Tsirka, 2012a).

## Granulocytes

Neutrophils are the leukocyte population that immigrates first into the brain after injury. In ICH, infiltrating neutrophils were found in and around the hematoma as early as 4 h after collagenase-induced ICH in mice (Wang and Tsirka, 2005). Their number peaks at 3–5 days after ICH (Gong et al., 2000; Xue and Del Bigio, 2000; Mracsko et al., 2014). Although the temporal pattern of neutrophil infiltration is similar in blood and collagenase injection models, higher neutrophil numbers are found after collagenase than after blood injection (Xue and Del Bigio, 2000; Mracsko et al., 2014). In experimental ICH, infiltrating neutrophils undergo apoptosis 2 days after entering the hematoma (Savill, 1997). Molecules released from dying leukocytes may further stimulate microglia/macrophages and exacerbate the neuroinflammatory process (Stern et al., 1996; Wang, 2010). Neutrophil accumulation in the blood vessels around the hematoma was observed already 6 h after the ictus (Wisniewski, 1961). In the peri-hematomal tissue obtained from ICH patients during craniotomy, neutrophil (and lymphocyte) infiltration further increased 12–24 h after ICH and correlated with the number of TUNEL positive cells (Guo et al., 2006). It should be noted however that in ischemic stroke histological techniques labeling components of the neurovascular unit showed that polymorphonuclear granulocytes were mainly located in the luminal surfaces or perivascular spaces of cerebral vessels and no granulocytes infiltrated the brain parenchyma (Enzmann et al., 2013). So far, no similar data are available for ICH.

Recent studies suggest an important role of activated microglia in neutrophil recruitment into the hemorrhagic brain. Heme-induced TLR4 activation on microglia increases CXCL2 production, which interacts with CXCR2 on the surface of neutrophils resulting in chemoattraction (Zarbock and Ley, 2009). Accordingly, TLR4-deficient mice show reduced neutrophil and monocyte infiltration 3 days after ICH (Sansing et al., 2011b).

Granulocytes appear to have mainly deleterious effects on the brain after ICH. Neutrophil depletion by intravenous injection of anti-polymorphonuclear neutrophil (anti-PMN) serum reduced BBB breakdown, axonal injury and neurological deficit (Moxon-Emre and Schlichter, 2011). After cerebral ischemia, anti-PMN therapy prevented endothelial dysfunction and thrombolysis-induced hemorrhagic transformation in another study (Gautier et al., 2009). As professional phagocytes, neutrophils use phagosomes containing digestive and oxidative compounds. During phagocytosis they produce an oxidative burst resulting in the release of ROSs via NADPH oxidase and myeloperoxidase (Hampton et al., 1998). Although these processes are needed for antimicrobial defence, high ROS levels due to microglial activation and neutrophil infiltration contribute to poor outcome after ICH (Nguyen et al., 2007; Han et al., 2008). The free radical scavenger edaravone decreases brain edema and neurological deficit after ICH (Nakamura et al., 2008). Other molecules with free radical trapping properties have been tested in ICH as reviewed earlier (Wang and Doré, 2007b) supporting the important role of ROS in secondary brain injury and their therapeutic potential after ICH.

Besides microglia/macrophages, the expression of the neurotoxic TNF-α has also been shown in neutrophils (Mayne et al., 2001b; Nguyen et al., 2007; Wasserman and Schlichter, 2007). Neutrophils may also recruit monocyte/macrophages amplifying inflammatory processes (Soehnlein and Lindbom, 2010). Accordingly, anti-PMN therapy decreased the number of infiltrating monocyte/macrophages around the hematoma and reduced glial scarring (Moxon-Emre and Schlichter, 2011).

## Cells of the adaptive immune system

Mounting an antigen-specific immune response generally requires several (5–7) days. As cellular parts of the adaptive immune system, B cells participate in humoral immune responses, while T cells are involved in cellular immunity. T cells express either the CD4 or the CD8 cell surface marker determining their function: modulating immune responses or eliciting cytotoxicity.

Increasing evidence suggests an important role of adaptive immunity and particularly T lymphocytes in secondary brain damage after ischemia (Yilmaz et al., 2006; Iadecola and Anrather, 2011; Liesz et al., 2011; Chamorro et al., 2012). In contrast, little is known about the role of lymphocytes after experimental and clinical ICH. Lymphocytes were found in cerebrospinal fluid early (starting at 6 h) following human ICH (Lee et al., 1975). Lymphocytes were also detected in peri-hematomal brain tissue obtained during craniotomy of ICH patients (Guo et al., 2006). In contrast, most studies using animal models of ICH reported more delayed infiltration of T cells 48–96 h after ICH (Xue and Del Bigio, 2000, 2003; Loftspring et al., 2009). Using flow cytometry, we found that CD4+ T cells are the predominating brain infiltrating leukocyte population in mice already 1 day after ICH and their number peaked at day 5 (Mracsko et al., 2014). At the same time, infiltration of CD8+ T cell appears to be less prominent in ICH compared to cerebral ischemia (Schwab et al., 2001; Loftspring et al., 2009; Chaitanya et al., 2010; Mracsko et al., 2014).

An important unresolved question is whether T cell invasion and activation is antigen dependent both in cerebral ischemia and in ICH (Iadecola and Anrather, 2011). Along with other T cell populations, both proinflammatory γδT cells and immunosuppressive regulatory T cells (Treg) infiltrate the hemorrhagic brain (Gao et al., 2014). According to the neuroprotective role of Treg in cerebral ischemia (Liesz et al., 2009), Treg transfer also attenuated neurological deficit after ICH (Yang et al., 2014b). The pathophysiological role of B cells and natural killer cells after ICH has barely been studied to date. Their low rate of infiltration (Mracsko et al., 2014) suggests a minor role in ICH-induced brain injury.

Fingolimod is a modulator of the sphingosine 1-phosphate receptor 1 and has been approved for the treatment of the relapsing form of multiple sclerosis. Fingolimod downregulates the expression of sphingosine receptors on T cells thereby inhibiting their egress from lymphoid tissue (Chiba, 2005). Fingolimod reduced brain edema and improved neurological function after experimental ICH in one study (Rolland et al., 2011). Interestingly, it was recently tested in a Chinese clinical pilot study (*n* = 23 patients) where it decreased peri-hematomal edema and reduced neurological impairment compared with control individuals (Fu et al., 2014). A better understanding of the mechanism of activation and action of T cell population is needed.

## Humoral inflammatory mediators

Nuclear factor-κB is a ubiquitous transcription factor that is a critical regulator of numerous responses including inflammation (Barnes, 1996) and pro-inflammatory genes such as TNF-α, IL-1β, nitric oxide synthase, HO-1 and intracellular adhesion molecule-1 (Barnes and Karin, 1997; Emsley and Tyrrell, 2002). Nuclear factor-κB has high sensitivity towards oxidative stress (Grilli and Memo, 1999) and gets immediately activated in the peri-hematomal brain tissue in both experimental (Hickenbottom et al., 1999; Wagner, 2007) and human ICH (Wang et al., 2011). Peroxisome proliferator-activated receptor-γ, a member of the nuclear hormone receptor superfamily has been shown to suppress NF-κB function leading to decreased inflammation and neuronal death, increased hematoma resolution and improved functional outcome after experimental ICH (Zhao et al., 2006). Information on the role of NF-κB in ICH has been reviewed elsewhere in detail (Aronowski and Hall, 2005; Wagner, 2007).

Cytokines can be divided into pro- and anti-inflammatory cytokines. Tumor necrosis factor-α is a pleiotropic cytokine which is mainly produced by microglia/macrophages (Lambertsen et al., 2005) and neutrophils (Mayne et al., 2001b). Tumor necrosis factor-α plays a central role in extending neuronal damage after CNS injury (Rodríguez-Yáñez and Castillo, 2008). Tumor necrosis factor-α knockout mice showed reduced ICH-induced brain edema compared to wild type mice (Hua et al., 2006). Treatment with TNF-α antibody after ICH attenuated microglia/macrophage activation, reduced cleaved caspase-3 and resulted in less brain edema and better neurological function (Mayne et al., 2001b; Lei et al., 2013). The IL-1 cytokine family contains an increasing number of members; the most important are IL-1α, IL-β and the natural receptor antagonist IL-Ra (Luheshi et al., 2009). In neuroinflammatory conditions, IL-1β is mostly produced by microglia/macrophages and is neurotoxic (Pearson et al., 1999; Vezzani et al., 1999). Overexpression of IL-1 receptor antagonist decreased thrombin-induced brain edema (Masada et al., 2001), BBB breakdown and neuronal loss (Greenhalgh et al., 2012). Both TNF-α and IL-1 are overexpressed as early as 2 h after experimental ICH (Xi et al., 2001b; Aronowski and Hall, 2005; Wagner et al., 2006).

Interferon-γ (IFN-γ) is one of the main effector molecule of T lymphocytes (Schroder et al., 2004) and T cells are the major source of IFN-γ in cerebral ischemia (Liesz et al., 2009). In contrast to the well-established expression pattern and role of IFN-γ in ischemic stroke (Yilmaz et al., 2006; Liesz et al., 2011), the role of this cytokine in ICH remains to be elucidated. Interferon-γ protein expression was increased at 72 h after ICH which was prevented by fingolimod treatment (Rolland et al., 2011).

Clinical studies on the role of cytokines in ICH are limited on serum measurements. Increased serum concentrations of IL-6 and IL-10 were found 24 h after ICH where IL-6 level correlated with blood volume and the mass effect of the hemorrhage (Dziedzic et al., 2002). In another study, elevated plasma levels of TNF-α and IL-6 12 and 24 h after the ictus correlated with peri-hematomal edema (Castillo et al., 2002). These reports support the deleterious effect of proinflammatory cytokines after ICH. Although cytokines may be promising therapeutic targets in ICH, to date no clinical trials examining the effect of cytokine antagonization have been conducted.

## Imaging of neuroinflammation

Due to the spatial and temporal complexity of the neuroinflammatory processes, anatomical and functional *in vivo* imaging techniques are increasingly recognized for diagnosis and follow-up in patient care. Furthermore, the fast development of these techniques already allows their implication for the understanding of neuroinflammatory mechanisms in the cellular and molecular level in experimental studies.

As microglia activation is an essential part of the neuroinflammatory response to cerebral injury and disease progression, it has become an important target for *in vivo* imaging of neuroinflammation. Upon activation, the microglial translocator protein (TSPO) is upregulated (Chauveau et al., 2008) and can be detected by radiolabeled ligands for positron-emission tomography (PET) or single-photon emission computed tomography (SPECT; Winkeler et al., 2010; Chauveau et al., 2011; Ciarmiello, 2011; Kiferle et al., 2011).

To investigate the mechanisms underlying the trafficking of systemic immune cells into the brain, contrast media targeting endothelial selectin, ICAM and VCAM have been developed. These include ^125^I-labeled gold nanorods (GdNRs) and 64Cu-labeled nanoparticles conjugated with anti-ICAM-1 antibody (Rossin et al., 2008; Shao et al., 2011) or iron oxide microparticles conjugated with anti-VCAM antibody (McAteer et al., 2007; Hoyte et al., 2010) (for detailed review cp. Jacobs and Tavitian, 2012)). Infiltrating leukocytes can be labelled either *ex vivo* by incubation with a tracer or *in vivo* taking advantage of their phagocytic properties. For *ex vivo* labelling ^111^In- or ^99m^Tc-labeled compounds for SPECT or [^18^F] fluorodeoxyglucose (FDG) for PET imaging have been developed (Wunder et al., 2009). *In vivo* labelling is performed using MRI agents including iron-oxide nanoparticles (Stuber et al., 2007), liposomes encapsulating monodisperse single core superparamagnetic iron-oxide particles (Soenen et al., 2010) or paramagnetic lanthanide-based agents (Castelli et al., 2009; Stoll and Bendszus, 2010).

The above detailed labeling methods are increasingly used in clinical and experimental studies to characterize inflammatory processes in neurologic disorders including cerebral ischemia, multiple sclerosis, Alzheimer’s and Parkinson’s disease (Jacobs and Tavitian, 2012). In contrast, *in vivo* neuroimaging has been barely used to investigate the ICH-induced inflammatory processes. In collagenase-induced ICH, enhanced MRI with microparticles of iron oxide targeted to VCAM-1 revealed the maximal VCAM-1 expression 24 h after ICH which returned to baseline 5 days following hemorrhage induction (Gauberti et al., 2013). However, so far we do not have neuroimaging data about tracking leukocytes infiltrating the hemorrhagic brain.

## Conclusions

Inflammatory processes are increasingly recognized as important players in the pathophysiology of secondary brain damage after ICH. There is now solid information on the infiltration pattern of leukocytes in experimental ICH. The pathophysiological role of specific leukocyte populations is beginning to be better understood but little is known about the interactions among these immune cells. Because of the delayed nature of brain damage after ICH, adaptive immune cells may play an important role in the subacute and the regenerative phases after ICH. Translation of preclinical findings into the clinical setting is challenging because of limitations of current animal models of ICH. Moreover, the local and systemic neuroinflammatory response in ICH patients remains to be better characterized.

## Authors’ contributions

All authors were involved in writing the review.

## Conflict of interest statement

The authors declare that the research was conducted in the absence of any commercial or financial relationships that could be construed as a potential conflict of interest.
